# Genetic Mutations Underlying Growth Impairment and Cardiomyopathies in Children: Molecular Mechanisms, Clinical Implications and Targeted Therapies

**DOI:** 10.3390/genes17030355

**Published:** 2026-03-23

**Authors:** Marco Maria Dicorato, Gaia De Sario, Maria Cristina Carella, Andrea Igoren Guaricci, Marco Matteo Ciccone, Cinzia Forleo, Gabriele D’Amato, Maria Felicia Faienza

**Affiliations:** 1 Cardiovascular Disease Section, Interdisciplinary Department of Medicine (DIM), University of Bari “Aldo Moro”, University Hospital Consortium, Polyclinic of Bari, 70124 Bari, Italy; m.dicorato20@phd.uniba.it (M.M.D.); m.carella31@phd.uniba.it (M.C.C.); andreaigoren.guaricci@uniba.it (A.I.G.); marcomatteo.ciccone@uniba.it (M.M.C.); cinzia.forleo@uniba.it (C.F.); 2 Giovanni XXIII Pediatric Hospital, University of Bari “A. Moro”, 70124 Bari, Italy; gaiadesario30@gmail.com; 3 Neonatal Intensive Care Unit, Di Venere Hospital, 70131 Bari, Italy; gabriele.damato@asl.bari.it; 4 Pediatric Unit, Department of Precision and Regenerative Medicine and Ionian Area, University of Bari “A. Moro”, 70124 Bari, Italy

**Keywords:** cardiomyopathy, growth, RASopathies, muscular dystrophies, Pompe disease, mucopolysaccharidoses, mitochondrial diseases, precision medicine, gene therapy

## Abstract

Growth impairment is a clinical manifestation frequently observed in pediatric patients with cardiomyopathy associated with various inherited disorders, including RASopathies, lysosomal storage diseases, neuromuscular disorders, and metabolic conditions. In this narrative review, we explored the genetic and pathophysiological mechanisms underlying the development of both growth and myocardial impairment in Noonan syndrome (NS)—the most common RASopathy—Duchenne and Becker muscular dystrophies, Pompe disease, mucopolysaccharidoses, and mitochondrial diseases. For each condition, we described the cardiac and growth phenotypes, focusing on epidemiology, clinical implications, and disease-specific therapeutic strategies. In the era of precision medicine, innovative etiologic treatments targeting the underlying molecular mechanisms have emerged. Therefore, elucidating the molecular pathways responsible for growth impairment in pediatric inherited cardiomyopathies remains essential for optimizing multidisciplinary management and improving patient outcomes.

## 1. Introduction

Cardiomyopathies (CMPs) are a group of heart muscle disorders characterized by structural, functional, and electrical impairment not explained by other loading conditions. In children, CMPs are less common than in adults but are associated with a high mortality and morbidity rate, including heart failure (HF) and heart transplantation [1,2]. In recent years, deeper insights into the genetic basis and molecular mechanisms of these pathologies have emerged due to continuous technological advancements and the spread of genetic testing. In most cases, children develop CMPs due to inherited disorders, associated with specific pathogenic variants in a wide range of possible genes [3]. Important data regarding genotype–phenotype correlations have been acquired over the decades. In the context of inherited CMPs, growth impairment, manifesting as short stature, is an important extra-cardiac feature that can be present in these individuals, whether or not it precedes the onset of the cardiac phenotype. Short stature is defined as a clinically significant reduction in linear growth. It is typically characterized by a height ≤ −2 standard deviations from the population mean, which corresponds to a value below the third percentile on age- and sex-specific standardized growth charts [4]. CMP and growth disorder are linked by the same molecular mechanism. The knowledge of this complex interplay is pivotal for personalized medicine and for tailored treatment that can address the primary etiology of the disease [5].

As in adulthood, the pediatric cardiac phenotypes range from dilated cardiomyopathy (DCM) and hypertrophic cardiomyopathy (HCM), which are the most common, to restrictive cardiomyopathy and arrhythmogenic cardiomyopathy [6]. As regards genetic etiology, the coexistence of cardiac and growth impairment is a feature mainly present in syndromic, metabolic, and neuromuscular diseases [1]. In this narrative review, we specifically focused on the most common inherited disorders in which both cardiomyopathy and growth impairment are recognized as clinical features, and for which sufficient clinical and molecular data are available to discuss shared pathogenic mechanisms and emerging or established therapeutic implications. In particular, we focused on Noonan syndrome (NS), the most prevalent RASopathy, distrophinopathies (including Duchenne and Becker muscular dystrophies), Pompe disease, mucopolysaccharidoses, and mitochondrial diseases. In such cases, a diagnosis may be made relatively late in the clinical course, particularly when short stature is the predominant clinical features. The heightened recognition of characteristic growth patterns has significant value for pediatricians, as it could potentially lead to earlier diagnoses. Earlier identification would, in turn, enable timely clinical management and appropriate genetic counseling for family members, especially those considering future pregnancies [7]. We also discussed in-depth treatment implications, including novel targeted therapies that could change the natural history of these patients.

## 2. RASopathies

RASopathies are a group of genetic disorders caused by mutations in genes encoding proteins involved in the RAS mitogen-activated protein kinase (MAPK) biochemical pathway. Half of cases are accounted for by a de novo mutation, whereas in the other half, an inherited mutation is found. The most frequently involved genes are represented by *PTPN11* and *SOS1*, even though more than 20 genes have been linked to RASopathy pathogenesis [8,9]. However, etiological mutations are not always found, remaining unknown in up to 20% of patients [10]. The RAS/MAPK pathway is a signaling cascade that regulates key cellular processes such as proliferation, differentiation, survival, and migration in response to extracellular stimuli [11]. In RASopathies, pathogenic variants cause pathway dysregulation by enhancing RAS activity or impairing its regulation. Some of the molecules involved include core RAS proteins (KRAS, HRAS, NRAS), regulators (SOS1/2, SHOC2), and downstream kinases (RAF, MEK, ERK) that transmit signals to the nucleus, with activating variants causing cascade hyperactivation [12]. Additional modulators such as PTPN11, LZTR1, and RIT1 further influence pathway intensity, with loss of negative regulation increasing RAS stability and signaling. Overall, excessive activation of the RAS/MAPK pathway disrupts normal development and underlies the diverse clinical features of RASopathies, with the degree of hyperactivation closely correlating with clinical severity and heterogeneity [13]. Distinct phenotypes reflect the specific gene affected, the strength of signaling activation, and tissue-specific effects of altered pathway function. A range of characteristic and almost pathognomonic features can be mentioned. They include short stature, congenital heart defects, facial dysmorphisms, and neurodevelopmental delay. Among these diseases, NS is the most common, with a prevalence that ranges from 1:1000 to 1:2500 individuals [11]. NS is characterized by a distinctive facial phenotype present from birth that evolves over time. Hallmark features in newborns include a disproportionately large head with a tall, broad forehead and hypertelorism with widely spaced eyes that is often accompanied by epicanthal folds and ptosis [14]. Other less common syndromes include neurofibromatosis, cardio-facio-cutaneous syndrome (CFCS) and Costello syndrome. In Costello syndrome, distinctive physical traits include full lips, a large mouth, a full nasal tip, curly or sparse fine hair, and loose and soft skin with deep palmar and plantar creases [15]. CFC syndrome has hallmark dermatologic manifestations, including poor hair growth, sparse hair on the arms and legs, sparse or absent eyebrows, and acquired melanocytic nevi, which can increase over time [16]. All these signs must be systematically investigated during the clinical evaluation, since they are mandatory for a prompt differential diagnosis [17]. Moderate activation of the RAS/MAPK pathway, as seen with PTPN11 variants, typically causes classical NS, whereas stronger activation from KRAS or BRAF variants leads to more severe phenotypes such as CFCS. Highly activating HRAS variants result in Costello syndrome, characterized by severe developmental delay, CMP, and increased cancer risk, while SOS1/2 variants are generally associated with milder or variable NS phenotypes [12,13]. NS-like RASopathies share a broadly similar spectrum of organ involvement with marked clinical overlap between and within conditions. Clinical expression is highly variable, and no single feature is pathognomonic of a specific RASopathy. Instead, all these disorders are primarily recognized by a characteristic combination of craniofacial features, cardiovascular anomalies, growth delay, and neurodevelopmental deficits, which represent the most connotative phenotypes [11]. These features result crucial for diagnosis and are linked to the same pathogenic molecular mechanisms described above.

### 2.1. Cardiac Phenotype

The cardiac involvement in RASopathies includes two main features: congenital heart diseases (CHDs) and HCM [10]. Genotype–phenotype correlations are well established, with PTPN11 variants mainly associated with pulmonary valve stenosis (PVS) and RAF1 variants strongly linked to HCM. Different variants within the same gene can lead to distinct RASopathies and cardiac outcomes, influencing prognosis and management [18]. The aberrant activation of RAS/MAPK enhances ERK phosphorylation, which is involved in the development of semilunar cardiac valves. Actually, PVS is the most common CHD in RASopathies, affecting about 40% of patients (70% of NS patients), followed by atrial and ventricular septal defects and atrioventricular canal defects [19,20]. On the other hand, RASopathies are a major cause of HCM in childhood, accounting for about 40% of infant HCM cases, with prevalence varying by syndrome. HCM is most frequent in Noonan syndrome with multiple lentigines (NSML), followed by Costello, CFCS, and NS, and is often severe with marked hypertrophy and left ventricular outflow tract obstruction (LVOTO) [21]. Mitral valve abnormalities, including systolic anterior motion of the anterior leaflet, anomalous chordal insertion, and papillary muscle displacement, frequently contribute to LVOTO and are associated with worse prognosis. They may also cause significant mitral regurgitation, leading to earlier onset of HF, with increased risk of reintervention and mortality [10]. Compared with non-syndromic HCM, RASopathy-associated HCM shows more complex anatomy, frequent bi-ventricular involvement, and arrhythmias, contributing to poorer outcomes [18]. Moreover, an earlier onset is reported compared to other pediatric HCM. In more than 50% of cases, it is diagnosed before six months of age [22]. Myocardial ischemia is present in up to 30% of patients and is usually related to coronary artery anomalies. Atrial arrhythmias are frequent, especially in Costello syndrome and RAF1 mutations [10]. Of note, arrythmias may be found even in the absence of structural heart changes [11,23]. Patients may also present complex cardiac phenotypes, combining CHD with HCM, with worse prognosis. Overall mortality in RASopathy-associated CHD is low, whereas HCM is a key prognostic factor associated with high mortality [10]. HF predominates as the cause of death in infancy, whereas sudden cardiac death becomes more relevant in adolescence [24].

### 2.2. Growth Impairment

The hyperactivation of the RAS-MAPK pathway leads to growth impairment due to a multifactorial and not completely understood process. Both altered growth hormone (GH) secretion and reduced peripheral response to GH are involved. Moreover, delayed puberty and hypogonadism seem to be implied in males [9]. A significant association between *PTPN11* gene mutation and short stature is well reported in the literature [25,26]. A study reported that GH secretion profiles, including baseline, mean and peak levels, were significantly higher in NS patients with PTPN11 variants compared to healthy children. PTPN11 encodes the SHP2 protein, which acts as negative regulator of the GH receptor. In the presence of PTPN11 mutations, SHP2 becomes hyperactive, leading to altered GH receptor response [27]. Growth in NS and related RASopathies is typically normal at birth. This is followed by postnatal growth deceleration, affecting up to 70% of patients with NS, often worsened by early feeding difficulties [28]. Delayed puberty and bone maturation further accentuate short stature during adolescence, although partial late catch-up growth may occur. Body proportions are generally normal, with relative or absolute macrocephaly being common [9,11]. The genotype has an important impact on stature. Growth failure is less marked in patients with NS caused by SOS1 mutations compared to those with mutations in other genes such as PTPN11, RAF1, and KRAS [25]. Patients affected by Costello syndrome show the most severe growth phenotype, reaching a height from 135 to 150 cm in adulthood [29]. Treatment with recombinant human growth hormone (rhGH) at the standard dose used for children with idiopathic GH deficiency effectively improves growth and adult height in NS patients with growth hormone deficiency. Additional studies are needed to evaluate the genotype-specific response to rhGH therapy across different pathogenic variants of the *PTPN11* gene and in less common genotypes [30].

## 3. Distrophinopathies

Muscular dystrophies represent a large group of inherited degenerative disorders that result in progressive muscle weakness due to intrinsic biochemical defects in a wide range of proteins, including extracellular, membrane and cytoplasmic proteins. Despite this heterogeneity, the presence of identical pathological changes in skeletal muscle has led to their classification as muscular dystrophies [31]. The genetic mutations that underlie many of the muscular dystrophies cause abnormalities not only in myocytes but also in a wide variety of other tissues [31,32,33]. Distrophinopathies are linked to mutations in the dystrophin gene, which is known to be the largest gene in humans. These diseases are characterized by a well-reported association of CMP, typically with a dilated phenotype, with a defect in growth of a multifactorial nature. Duchenne muscular dystrophy (DMD) is the most prevalent childhood muscular dystrophy, with a prevalence of approximately 1:5000 boys [32]. Becker muscular dystrophy (BMD) represents a milder allelic variant of DMD [34]. The etiology of both conditions is attributed to mutations in the dystrophin gene, with an X-linked recessive inheritance pattern being observed in affected individuals. Distinct clinical manifestations are the result of differences in dystrophin (Dp) expression and functional status [33]. The primary phenotypic distinction between DMD and the milder BMD is attributed to the “reading frame rule.” Actually, mutations that disrupt the open reading frame result in the production of a nonfunctional, truncated dystrophin, thereby leading to a more severe phenotype (DMD). In contrast, mutations that maintain the reading frame yield a shorter, yet partially functional protein, causing BMD [32]. Thus, dystrophin is nearly absent in DMD, while it is partially functional in BMD. The large size of the DMD gene renders it more susceptible to mutations, with approximately one third of them arising de novo [33,35]. A wide range of mutations are possible, most of which are represented by deletions. Dystrophin is part of a large transmembrane complex, where it is located on the membrane surface of skeletal and cardiac myocytes. There, it serves an essential function by connecting the cytoskeleton to the extracellular matrix, preserving membrane integrity [32]. A truncated and unstable dystrophin results in disruption of cell survival pathways and cellular defense mechanisms, thereby increasing the susceptibility of cells to undergoing apoptotic and necrotic death [31]. It plays a pivotal role in reinforcing the sarcolemma and protecting muscle fibers from contractile stress. The dystrophin protein is expressed not only in skeletal muscle but also in other tissues, including the brain, retina, and smooth muscle. Therefore, its deficiency contributes to the wide spectrum of clinical manifestations observed in patients with DMD and BMD [31].

### 3.1. Cardiac Phenotype

The development of cardiac dysfunction is a hallmark of DMD patients, manifesting in nearly all cases, above all if the patient has surpassed the age of 18 years [36,37]. Moreover, the enhanced survival rates attributable to advancements in respiratory therapy have resulted in an augmented prevalence of cardiomyopathy [38]. The cardiac phenotype typically manifests as progressive DCM during adolescence, accompanied by progressive myocardial scarring [39]. The influence of specific dystrophin mutations on the risk of CMP and the response to therapeutic interventions is a subject of scientific investigation. Electrocardiographic (ECG) abnormalities frequently precede structural changes and reflect myocardial fibrosis patterns. The most prevalent of these are Q waves in the left precordial leads, right axis deviation, and right bundle branch block. Diastolic dysfunction, typically linked to dystrophin-related calcium dysregulation, is often identified at an early stage. This state of “tonic contraction” frequently occurs prior to DCM [40]. The progressive non-ischemic cardiac fibrosis originates in the subepicardic posterobasal segment of the left ventricle (LV) and subsequently disseminates throughout the myocardium and septum. These patterns can be readily detected by cardiac magnetic resonance (CMR) through late gadolinium enhancement (LGE) [41]. Fibrosis manifests with a heterogeneous distribution, ultimately resulting in regional wall motion abnormalities, ventricular thinning, loss of contractility, and dilated cardiomyopathy [39,42]. In contrast with other CMPs, LV dilation is relatively modest despite severe functional impairment. The clinical features manifest as a reduction in fractional shortening, a decrease in left ventricular ejection fraction (LVEF), an increase in ventricular volumes, and mitral regurgitation. Despite the presence of fibrotic and calcific changes in the right ventricle (RV), RV systolic function is frequently maintained [43,44]. Diffuse fibrosis has been shown to promote ventricular remodeling; in contrast, focal fibrosis has been demonstrated to increase the risk of arrhythmias and sudden death. Indeed, arrhythmias are prevalent in DMD and are the result of altered calcium handling, oxidative stress, and ion channel dysregulation, in addition to myocardial failure [45]. As people age, both atrial and ventricular arrhythmias increase, and they correlate with declining systolic function and extensive fibrosis [46]. Life-threatening arrhythmias, including ventricular tachycardia and fibrillation, occur in advanced disease [47,48]. Furthermore, the diagnosis of DMD-related CMP is complicated by the limited physical activity exhibited by these patients and the overlapping respiratory symptoms that are present in them. Consequently, periodic cardiac surveillance visits, encompassing ECG, Holter ECG, and echocardiography, are imperative [49].

Since both DMD and BMD are X-linked disorders, they primarily affect males. However, although female carriers possess one normal copy of the gene, they may still develop muscular weakness and heart manifestations [50]. The main molecular mechanisms, despite not being completely understood, are represented by chromosomal aberrations, inheritance patterns (such as compound heterozygotes or consanguinity), and hormonal events [51,52]. In particular, cardiac involvement is not infrequent in this population. DCM has been reported in up to 17% of female DMD carriers and up to 13% of female BMD carriers [53]. Symptoms onset ranges from childhood to adulthood, most often around puberty. The likelihood and severity of DCM increase with age and may progress to advanced heart failure, occasionally requiring heart transplantation [54]. Arrhythmias or conduction defects may occur as well. Cardiac involvement can be present even without skeletal muscle symptoms or become evident during stressors such as pregnancy or anesthesia [53].

### 3.2. Growth Impairment

The DMD phenotype is characterized by the presence of restrictive pulmonary disease, DCM, scoliosis, and complications associated with prolonged corticosteroid therapy [32]. Glucocorticoid therapy represents the most prevalent treatment modality, and it is currently the sole therapy that has demonstrated consistent benefits, including a two-year increase in ambulation duration and the preservation of cardiorespiratory function. However, it should be noted that long-term use of these medications has been associated with a number of significant adverse effects, including but not limited to osteoporosis-related fractures, impaired linear growth, and delayed puberty [34]. Glucocorticoids have been demonstrated to impede growth through several mechanisms, including toxicity to growth plates, inhibition of differentiation in both chondrocytes and osteoblasts, and the induction of their apoptosis [55]. Furthermore, these substances have been observed to reduce GH release by increasing hypothalamic somatostatin and decreasing pituitary sensitivity to GH-related hormone. This results in lower levels of both GH and insulin growth factor-1. A study of DMD patients treated with glucocorticoids revealed that approximately 45% of the subjects exhibited reduced peak GH responses upon stimulation. With regard to the utilization of glucocorticoids, growth impairment appears to be influenced by several factors, including the duration of glucocorticoid therapy, the dosing regimen (daily versus intermittent), and the specific agent employed (prednisone versus deflazacort). The extant literature, comprising both observational studies and clinical trial data, indicates that daily regimens, as well as the use of deflazacort, are associated with greater growth delay compared to intermittent dosing and prednisone [56,57]. A preponderance of research findings suggests that the manifestations of reduced growth and short stature are prevalent even among glucocorticoid-naïve patients, with adult height typically exhibiting a standard deviation below the mean. However, this phenomenon is more pronounced in cases where steroid therapy is administered. The mechanisms underlying growth impairment in DMD remain to be elucidated [57]. Recent evidence suggests that mutations in the distal segment of the dystrophin gene, which are predicted to disrupt Dp71 expression, may affect height regulation, suggesting a possible correlation between dystrophin genotype and growth. Nevertheless, the precise mechanism by which altered Dp71 expression contributes to growth failure remains poorly understood [55]. Conversely, the mechanisms underlying growth impairment in BMD are hypothesized to be analogous to those observed in DMD. In previous years, BMD has predominantly existed in a state of relative obscurity, largely regarded as a discrete entity rather than a component of a unified condition with a variable phenotype. Consequently, the extant literature on BMD is limited [34,56].

## 4. Storage Diseases

### 4.1. Pompe Disease

Glycogen storage diseases (GSDs) are a group of inherited metabolic disorders that affect multiple organ systems. They are characterized by the accumulation of glycogen due to deficiencies in the enzymes responsible for its synthesis or breakdown. The various forms of GSD have been assigned numerical designations based on the chronological order in which their enzymatic defects were identified. The liver and muscles, which contain the highest glycogen reserves, are the most severely affected tissues. However, the heart, kidneys, and brain can also be involved [58]. The most acknowledged GSD, in which an overlap between CMP and growth impairment is relatively common, is Pompe disease (PD). PD is a rare, autosomal-recessive genetic condition, also referred to as GSD type 2 or acid maltase deficiency. The incidence of PD has been estimated at approximately 1:40,000 live births. However, the recent implementation of newborn screening has revealed a much higher prevalence [59]. The etiology of this condition is attributed to mutations in the GAA gene, which is located on chromosome 17. This gene encodes acid α-glucosidase (GAA), a lysosomal hydrolase. Mutations in the GAA gene result in a deficiency of the GAA enzyme and progressive accumulation of glycogen within lysosomes of multiple tissues, particularly in cardiac and skeletal muscle. A total of 500 GAA mutations has been identified, encompassing insertions, deletions, splice-site, nonsense, and missense mutations. GAA is synthesized as a precursor and is directed to the lysosomes, where, through proteolytic processes, it is transformed into the mature, active enzyme [60,61]. In patients diagnosed with PD, the defective enzyme is incapable of catalyzing the hydrolysis of the α-1,4 and α-1,6-glucosidic bonds in glycogen, resulting in its accumulation in lysosomes. This accumulation leads to cellular dysfunction and muscle damage [62]. The clinical expression is determined by the specific GAA mutations and the resulting level of GAA enzyme activity. The wide range of phenotypic manifestations encompasses variability in organ involvement, severity of clinical symptoms, and age of onset. The clinical forms of the disease include two main categories: the most severe infantile-onset Pompe disease (IOPD), which is further classified into classical (CIOPD) and non-classical (NCIOPD) subtypes, and the milder late-onset Pompe disease (LOPD) [63]. IOPD is characterized by the presence of severe symptoms, including CMP, with onset before 12 months of age. In CIOPD, the most pathognomonic clinical feature at birth is the combination of marked hypotonia (“floppy infant”) and severe HCM [64]. In contrast, LOPD encompasses all individuals with the same early onset but without cardiomyopathy, as well as those with onset after 12 months of age [65]. In this form of the disease, common findings include proximal muscle weakness and progressive respiratory insufficiency [66]. Conversely, presentation with limb–girdle muscle weakness is more typical of muscular dystrophies [67]. A distinguishing feature of PD is that respiratory insufficiency can manifest early, even while patients are still ambulant. In contrast, in other neuromuscular disorders, respiratory involvement ordinarily manifests after the loss of ambulation [68]. The variability in clinical presentation is attributed to the level of GAA activity. Patients with IOPD generally exhibit an almost complete absence of enzyme activity (<1%), leading to severe hypotonia, generalized muscle weakness, feeding difficulties, failure to thrive, cardiomyopathy, and respiratory distress. These symptoms are often fatal if untreated. In contrast, individuals with LOPD exhibit partial GAA activity, resulting in a milder and more slowly progressive disease course [62].

#### 4.1.1. Cardiac Phenotype

Cardiac involvement in CIOPD is virtually universal and is characterized by a severe HCM phenocopy due to the progressive storage of glycogen in myocardial cells. The onset of HCM is very early and can be present before birth, as well. If not treated, PD-related CMP evolves rapidly into end-stage DCM, with a decrease in LVEF and advanced heart failure. Exitus occurs within the first two years of life [69]. The most prevalent ECG features identified are a short PR interval and signs of LV hypertrophy. Echocardiography is a fundamental diagnostic procedure that can reveal various cardiac abnormalities related to HCM, including bi-ventricular thickening, primarily in the interventricular septum and the posterior left ventricular wall [70]. It can also demonstrate reduced volumes and diastolic dysfunction. LVOTO, although not prevalent, has the potential to complicate the management and prognosis of patients with this condition [71,72]. A recent Chinese study demonstrated that LVEF, E/e’ratio, maximum LV wall thickness, LV posterior wall, LVOTO, and global longitudinal strain were independently correlated with survival [73]. In addition, glycogen deposits induce conduction disorders, ventricular arrhythmias, and subendocardial ischemia due to inadequate coronary perfusion, which can ultimately result in sudden cardiac death [74]. In CIOPD, cardiomegaly and massive ventricular hypertrophy are frequently present at diagnosis and may even be detected in the neonatal period [75]. CMR has been studied as an important imaging tool to quantify LV mass index and EF. It provides additional information on myocardial structure, even though LGE is not often present. This technique showed promising results even in monitoring treatment response in children with CIOPD over time [76].

In NCIOPD, cardiac impairment has been reported in approximately 10% of cases, manifesting as a less severe phenotype [77]. A recent systematic review of NCIOPD subjects revealed an impairment in LV global radial and circumferential strain, as measured by CMR. ECG abnormalities manifested in a manner analogous to those observed in classical forms, albeit with a reduced prevalence, occurring in approximately 10% of cases [78]. Notable features found in the investigation were various valvulopathies, LV hypertrophy, and lower LVEF occurring in these individuals. It is noteworthy that these conditions manifested in a limited proportion of the NCIOPD population, with prevalence ranging from 1 to 21%, contingent upon the specific feature under consideration. Wolff–Parkinson–White syndrome (WPWs), HCM with LVOTO, and DCM have rarely been described [79]. Cardiac manifestations are rarely observed in adults due to the presence of partial enzymatic activity. In instances where it is present, its onset occurs in the third decade of life. In such cases, DCM has also been documented, and endomyocardial biopsy is frequently indicated for diagnostic purposes. The occurrence of arrhythmias is an infrequent phenomenon; however, cases of WPWs have been documented [80].

#### 4.1.2. Growth Impairment

Children affected by PD are at elevated risk of precocious puberty, with documented rates of up to 75% in female patients and 40% in male patients. The majority of PD patients with precocious puberty have a significantly reduced adult height. Precocious puberty has been shown to accelerate bone maturation and lead to early epiphyseal closure, resulting in a reduction in adult height. The precise mechanisms underlying this phenomenon remain poorly understood; however, one hypothesis posits that lysosomal glycogen accumulation within the hypothalamic–pituitary–gonadal axis may play a role. In a recent study involving 19 PD patients, brain magnetic resonance imaging did not reveal any lesions in the hypothalamic–pituitary region. The only abnormality observed consisted of varying degrees of cortical hypomyelination. Hypomyelination may be considered an indirect consequence of cellular damage caused by glycogen accumulation [81]. Thus, growth deficit in PD is not solely associated with chronic muscle weakness or illness but also with alterations in pubertal timing. This underscores the necessity for comprehensive monitoring of growth and puberty even in patients receiving long-term therapy [82]. In children diagnosed with LOPD, growth is typically within the normal range. However, some patients present with mild feeding difficulties or dysphagia, which underscores the importance of dietary adjustments, such as a reduced carbohydrate intake and increased protein consumption, in preventing potential complications [83].

### 4.2. Mucopolysaccharidosis

Mucopolysaccharidoses (MPSs) are a group of inherited lysosomal storage disorders caused by deficiencies of enzymes involved in the degradation of glycosaminoglycans (GAGs). GAGs are complex carbohydrate molecules that are widely distributed in connective and other tissues throughout the body. The progressive GAG accumulation within lysosomes leads to multisystem clinical complications [84,85]. The incidence of MPSs varies widely across countries, ranging from 1.35 to 16.9 in 100,000 live births [86]. These conditions are classified into seven distinct types, each defined by a particular enzyme deficiency and distinct GAG accumulation. The majority of forms exhibit autosomal-recessive inheritance, with the exception of MPS type II (Hunter syndrome), which demonstrates X-linked inheritance [87]. A notable overlap exists among the systemic manifestations exhibited by the various subtypes of MPS, although each individual subtype is distinguished by specific predominant features. Typically, MPSs are asymptomatic at birth, with clinical manifestations emerging in early childhood. MPS type I and type II are the most prevalent forms. Typically manifesting in early childhood, severe cases are distinguished by systemic complications, including cognitive impairment [88]. Severe forms of MPS type I manifest with dysostosis multiplex, hearing loss, and intellectual disabilities, while attenuated forms may present with late-onset learning difficulties [89]. Cutaneous manifestations are typical of MPS type II, sometimes even representing the first manifestation of the disease [90]. MPS type III is distinguished by neuropsychiatric symptoms, including severe intellectual disability and developmental regression [88]. MPS type IV is primarily characterized by skeletal dysplasia, occurring in early childhood and usually accompanied by short stature [91]. MPS VI is caused by a deficiency of the enzyme arylsulfatase B, leading to an accumulation of dermatan sulfate and chondroitin sulfate. The condition manifests with multisystem features reminiscent of those observed in MPS I and II [92]. MPS VII may present perinatally with some typical features, including nonimmune hydrops fetalis, cholestatic jaundice, early organomegaly, and, in severe cases, early death. In childhood, the most suggestive elements are represented by dysostosis multiplex and coarse facial features, including macrocephaly, macroglossia, and corneal clouding [93]. According to previously published data, the diagnosis of these patients is made at a later age, and a higher incidence of cardiac abnormalities has been observed [94]. MPS VII is an exceedingly rare condition, with affected patients presenting with nonimmune fetal hydrops, a relatively distinctive clinical feature of this condition [86].

#### 4.2.1. Cardiac Phenotype

Cardiac involvement has been documented as a possible feature of MPS, particularly in types I, II, and VI, in which its prevalence has been reported to reach up to 80% of cases [95,96]. Conversely, its occurrence is less frequent in the other forms [86]. GAGs are deposited in every structure of the heart, resulting in valvular disease, arrhythmia, coronary artery disease, and CMP. The most thoroughly documented features are represented by valvular abnormality and valvular heart disease typically involves progressive thickening and dysfunction of the mitral and aortic valves, resulting in combined valvular stenosis and regurgitation. LV hypertrophy is frequently concentric and may reflect both myocardial infiltration and chronic pressure overload secondary to valvular disease, with reduced ventricular compliance [97]. Coronary artery disease is characterized by diffuse intimal proliferation and luminal narrowing, contributing to myocardial ischemia and sudden cardiac death [98]. Conduction system abnormalities include atrioventricular block, bundle branch block, and supraventricular arrhythmias [99]. HCM occurs in approximately 50% of MPS-I patients [100]. Documented cases of DCM have also been reported [101]. MPS-related CMP manifests predominantly during early life and progresses over time. The onset of the syndrome in infancy has also been reported in conjunction with acute HF [95]. Patients with severe forms (MPS I and II) exhibit earlier and more severe cardiac impairment compared to attenuated forms [102]. The advent of CMR has facilitated more precise characterization of myocardial involvement, even in a precocious phase of disease. However, evidence in the setting of MPS still lacks [103]. The presence of early cardiac involvement has been demonstrated to be associated with a worse prognosis. Indeed, approximately 50% of MPS patients succumb to cardiac causes [104]. Early diagnosis of MPS is paramount for ensuring prompt cardiac evaluation.

#### 4.2.2. Growth Impairment

Skeletal involvement is a common feature across most MPS types, resulting from GAG accumulation in bone and cartilage. This frequently leads to growth impairment that worsens with age. Actually, the accumulation of GAGs instigates inflammatory pathways and lipid signaling, which in turn propel cartilage apoptosis, synovial hyperplasia, and skeletal abnormalities [105]. Specifically, studies have demonstrated that synovial fibroblasts in MPS patients exhibit a marked upregulation of Tumor Necrosis Factor, reaching up to 50-fold, which results in an increase in the production of osteoclast survival factor. The subsequent formation of multinucleated osteoclast-like cells results in skeletal abnormalities [106]. Growth phenotypes vary among MPS syndromes, depending on the type and number of GAGs accumulated and generally reflecting disease severity. Growth in all MPS diseases differs significantly from the general population [107]. Growth velocity in patients with MPS I decreases after the age of 2 years [105]. Patients with MPS II often present with disproportionate stature due to skeletal dysplasia, with a markedly reduced final adult height [108]. Growth impairment in MPS II occurs independently of cognitive involvement or disease severity [109]. In MPS III, growth remains normal until around 6 years of age. In these forms, there is a correlation between adult height and disease and neuropathy severity, unlike MPS II [110]. Patients with type IVA MPS usually manifest greater length and weight compared to the general population. This is probably attributed to connective tissue laxity, allowing greater body extension at birth. Nevertheless, MPS-IVA exhibits the most severe growth impairment, with children growing slowly after birth and reaching a final height equivalent to that of a typical 7-year-old [111]. MPS VI follows a similar pattern, whereas in MPS I, II, and III, growth deceleration occurs later [112]. MPS III shows the mildest impact on growth [113].

## 5. Mitochondrial Diseases

Mitochondrial diseases (MDs) include a wide cohort of rare syndromic conditions caused by mutations in genes involved in mitochondrial function. The collective incidence is approximately one in 5000 live births, with a bimodal distribution during early infancy and in early adulthood [114]. They are characterized by a high degree of complexity, exhibiting diverse inheritance patterns. Mutations may be classified as maternal, since they are transmitted through mtDNA, or autosomal (recessive or dominant) or X-linked when arising from nuclear genes. De novo mutations, without a familiar basis, are also possible [115]. Each human cell contains multiple copies of mitochondrial genome (mtDNA), and pathogenic variants may coexist with normal mtDNA in varying proportions, a phenomenon known as heteroplasmy. In case of mtDNA variants, the onset of disease is precipitated by the accumulation of mutant mtDNA that surpasses tissue-specific thresholds, particularly in energy-dependent organs, such as brain, muscles, and the endocrine system. It is well acknowledged that 37 genes are present in the mtDNA, but nuclear genes are responsible for encoding the majority of mitochondrial proteins, which are synthesized in the cytosol and subsequently imported into the mitochondria [116]. Advancements in next-generation sequencing have led to the rapid expansion of gene discovery, with nearly 400 genes now associated with primary MD. In adult patients, MDs are more often attributable to pathogenic variants in mtDNA, while in pediatric cases, nuclear DNA mutations predominate [117]. Some of the genes involved in pediatric MD are represented by genes of OXPHOS complexes (NDUFS1, NDUFS2, NDUFV1, NDUFAF), genes implied in mtDNA maintenance (POLG), mtDNA depletion (TWNK and MPV17), and mitochondrial processes (DNM1L). These pathogenic variants finally affect a multitude of proteins that are implicated in mitochondrial biochemical pathways, leading to possible defects in (1) the OXPHOS system, (2) mt DNA maintenance, (3) mt gene expression, (4) cofactor biosynthesis, (5) mt molecule transport, (6) mt lipid membranes and organellar processes [115]. The result is a loss of normal energy production in cells of different tissues. The pathogenesis of MD is mainly but not solely linked to this mechanism of energy deficiency; indeed, other pathogenic processes are represented by disrupted signaling and redox balance, calcium homeostasis, and apoptosis. The expressivity of MD is also influenced by environmental and genetic modifiers, and even individuals with identical mutations may exhibit significant phenotypic variability. This clinical heterogeneity is even more present in children, who often do not present with classic syndromes, particularly in early stages, when only a single organ may be affected [115]. Each MD has some hallmark clinical features, which are usually summarized in the acronym name of the pathology. These distinctive signs and symptoms, when found together, become almost pathognomonic. Mitochondrial Encephalomyopathy with Lactic Acidosis and ‘Stroke-like’ episodes (MELAS) is characterized by Stroke-like episodes that do not follow vascular territories on imaging, lactic acidosis, myopathy, short stature, hearing loss, and diabetes [118]. Mitochondrial Encephalomyopathy with Ragged red Fibers (MERRF) typically presents with myoclonus and epilepsy, even though the pathognomonic finding is represented by ragged-red fibers on muscle biopsy. Progressive External Ophthalmoplegia is a distinctive clinical element of Chronic Progressive External Ophthalmoplegia (CPEO) and Kearns–Sayre syndromes (KSS), along with diabetes [119].

Barth syndrome is an X-linked recessive disorder, resulting from mutations in tafazzin gene (TAZ), which lead to impaired cardiolipin remodeling and consequent mitochondrial dysfunction [120]. Because of the X-linked inheritance, clinical manifestations develop almost exclusively in males and include CMP (above all, DCM or HCM, and LVNC), skeletal myopathy, neutropenia, and growth delay [121]. Female carriers are usually asymptomatic because skewed X-chromosome inactivation tends to favor cells expressing the normal TAZ gene [122]. However, rare symptomatic female cases have been reported when additional chromosomal abnormalities affect the X chromosome carrying the normal gene [123]. In these very uncommon situations, reduced tafazzin production may result in a clinical phenotype similar to affected males, including both CMP and growth impairment [124].

### 5.1. Cardiac Phenotype

Mitochondrial dysfunction has been identified as a primary contributing factor to the development of heart failure, with studies indicating its role in the onset of energetic deficits and oxidative stress [125]. These phenomena, in turn, have been linked to the occurrence of cardiac remodeling and contractile impairment. The presence of elevated mitochondrial ROS exacerbates arrhythmias, hypertrophy, and cell death. Collectively, these mechanisms establish a vicious cycle that leads to CMP in MD [117,126]. Cardiac involvement in MD is reported in up to 40% of patients, above all in individuals bearing large-scale mtDNA deletions or the m.3243A>G mutation [127]. In children, the most acknowledged MDs that involve the heart are Barth and Sengers syndromes but also Leigh syndrome, Friedrich’s ataxia and MELAS [115,117]. Cardiac involvement is highly heterogeneous, with phenotypes ranging from asymptomatic ECG changes to severe heart failure, fatal arrythmias and sudden cardiac death. The most typical presentation is represented by HCM phenocopy; however, unlike sarcomeric HCM, hypertrophy is typically concentric and in advanced phases may evolve into dilation, with a loss of systolic function. Asymmetric LV, right ventricular involvement, and primary DCM have been described too. Hypertrabeculation of the LV is especially common in pediatric-onset MD, sometimes fulfilling criteria for left ventricular noncompaction [117]. Conduction defects with even advanced heart block may be present, above all in KSS [128]. Increased LV mass and LV mass/volume ratios can be accurately detected by CMR [129]. LGE imaging often demonstrates a non-ischaemic pattern of intramural or subepicardial fibrosis, most commonly involving the basal inferolateral segments, which may reflect myocardial replacement fibrosis from chronic energetic dysfunction. However, LGE distributions are heterogeneous, especially comparing MELAS and CPEO/KSS phenotypes [130]. Beyond morphology, advanced techniques such as T1 and T2 mapping and extracellular volume quantification show promise for differentiating MD from other HCM phenocopies (e.g., amyloidosis, Fabry disease) and tracking disease progression [131].

### 5.2. Growth Impairment

A wide range of endocrine disorders is possible in MD, ranging from diabetes mellitus, which is the most frequent, to GH deficiency, hypogonadism, adrenal dysfunction, and thyroid and parathyroid disease. Growth impairment is not uncommon in these patients and has been linked mainly to cytochrome c oxidase (complex IV) deficiency, such as in Leigh syndrome [132]. Short stature is observed in up to 50% of MELAS individuals and from 40 to 90% of those affected by Leigh syndrome and KSS [7]. Growth impairment combined with CMP may occur in virtually all MDs and is more typical of dilated cardiomyopathy and ataxia, Barth syndrome, Pearson syndrome and KSS [117,133]. In these conditions, the coexistence of cardiac dysfunction and metabolic inefficiency may further aggravate growth delay through increased metabolic demand and reduced exercise tolerance. Past studies have demonstrated how MD children often manifest short stature and are relatively thin, also exhibiting a lower Body Mass Index (BMI). These features persist in adulthood and may follow a progressive trajectory parallel to disease severity [7]. Short stature is frequent in patients affected by MELAS, in particular, the juvenile-onset form, sometimes preceding the onset of neurological manifestations. Growth failure in MD stems from a multitude of interacting mechanisms, including inadequate nutrition, defective substrate utilization, and insufficient energy production. The decline in BMI suggests that poor nutritional status is a key contributor, supporting nutritional optimization rather than excessive caloric intake. The presence of feeding difficulties, including dysphagia and vomiting, in conjunction with neuromuscular involvement, further compromises growth in early childhood. Lastly, reduced physical activity related to myopathy and fatigue may alter body composition and contribute to reduced lean mass accumulation [134]. Mitochondrial dysfunction has also been demonstrated to affect fetal development, resulting in low birth weight. GH deficiency has also been described in the context of mtDNA deletion syndromes and MELAS. However, a considerable number of patients with short stature do not manifest GH deficiency. Hypothalamic–pituitary axis dysfunction has been proposed as a possible underlying mechanism of GH abnormalities. Indeed, the efficacy of GH therapy in promoting linear growth has been demonstrated to vary, and it should be administered with caution, accompanied by meticulous clinical monitoring [7,132,133,135] (Table 1).

## 6. Etiological Therapies and Future Directions

The advent of precision medicine has given rise to novel treatment strategies across various medical disciplines and in the domain of genetic diseases. The advent of precision medicine, marked by the ability to target specific pathogenic biochemical pathways and specific DNA sequences, has precipitated substantial changes in the management of such genetic conditions, profoundly altering their natural history [137]. Recent therapeutic interventions have demonstrated efficacy in addressing not only growth impairment but also cardiac involvement, which is frequently the primary contributor to patients’ prognoses. Nevertheless, etiologic therapy remains elusive for the preponderance of these conditions [138]. Scientific research is undergoing continuous growth, and novel molecules capable of acting at the precise etiology of the disease are in the development stage. This advancement is contingent upon a comprehensive understanding of the precise biochemical pathways and genetic defects that underpin these pathologies.

### 6.1. RASopathies

The clinical management of RASopathies has historically prioritized the treatment of cardiovascular complications, particularly HCM and structural heart defects. This approach reflects longstanding gaps in knowledge regarding the precise molecular mechanisms of these diseases, also driven by their high heterogeneity due to complex gene interactions, downstream signaling responses, and environmental influences. However, therapeutic manipulation of the RAS–MAPK and PI3K–AKT signaling pathways poses significant challenges, as they are critical to normal cellular function and development. Their widespread expression and marked interconnectivity of the RAS signaling networks limit the effectiveness of strategies aimed at blocking a single molecular target [139]. This signaling specificity underscores the necessity of pathway-tailored—and ultimately mutation-specific—treatment strategies for RASopathies. Among pathway-targeted agents, MEK inhibitors represent the most advanced therapeutic class for RASopathies. Selumetinib became the first FDA-approved treatment in this group following its approval for children with NF1-associated inoperable plexiform neurofibromas [140]. Additional clinical experience, primarily from case series, suggests that Trametinib can improve HCM in NS, even with some evidence of improved growth outcomes [141]. Other downstream pathway inhibitors, including pan-RAF and ERK inhibitors, are under investigation but limited by tolerability concerns. However, MEK inhibition does not have universal efficacy across the RASopathy spectrum. In disorders such as NSML, where pathogenic signaling is primarily driven by PI3K–AKT hyperactivation rather than MAPK dysregulation, alternative therapeutic approaches with inhibitors targeting mTOR, AKT, or upstream tyrosine kinases successfully reversed HCM [12]. Treatment with the mTOR inhibitor Everolimus stabilized severe cardiac disease in an infant with NSML [142]. Some studies demonstrated the efficacy of the tyrosine kinase inhibitor Dasatinib in reversing the cardiac phenotype in NS mice [143]. Despite this promising data, the routine clinical use of targeted therapies for RASopathies remains limited and clinical evidence in humans largely consists of individual case reports, underscoring the need for well-designed prospective trials.

### 6.2. Distrophinopaties

The development of etiologic therapies for DMD is complicated by extensive mutation heterogeneity, limiting the efficacy of mutation-specific treatments. Moreover, the large size of dystrophin gene poses technical challenges too. Novel effective treatments are emerging that target the primary molecular defect in DMD, including gene editing, exon skipping, stop codon readthrough, and viral delivery of truncated dystrophin. Stop codon readthrough strategies target nonsense mutations, which represent approximately 10% of cases. The translational readthrough of premature stop codons is promoted by small molecules, which restore production of full-length dystrophin [144]. Exon skipping targets mutation “hot spots” in the dystrophin gene, based on the reading frame rule. This principle has been exploited to convert DMD mutations into a Becker-like phenotype. Antisense oligonucleotides (AONs) bind to splicing sites to exclude specific exons from the mature mRNA. AONs able to realize the skipping of exon 51, 53 and 45 have gained FDA approval, though their clinical benefit and population treated remain limited [145]. Because exon skipping is temporary, repeated administration is required. Future strategies aim to enhance AON efficacy and enable multi-exon skipping, with potential benefit for a larger proportion of patients. Direct replacement of dystrophin by gene therapy is challenging because of the protein’s large size and the limited packaging capacity of adeno-associated virus (AAV) vectors (insufficient for full-length Dp), with possible immune responses [146,147]. Observations from BMD patients led to the design of truncated “microdystrophin” constructs that can be delivered by AAV and correctly localize to the muscle membrane. These microgenes are functional compromises compared with full-length dystrophin, that have shown encouraging results. However, this treatment is limited by necessity of repeated administrations and critical timing of treatment due to muscle growth and degeneration [148,149]. Muscle-specific promoters play a cornerstone role in preventing immunogenicity and improving efficiency. Among them, MHCK7 has been used for delandistrogene moxeparvovec, which represents the first gene therapy approved by the FDA for DMD in 2025 [147]. Several other gene therapies are in the first phase of study [150]. CRISPR/Cas9-mediated genome editing represents a promising alternative under investigation. Guided by a gRNA, Cas9 creates a double-strand break in target DNA, which can be repaired via error-prone non-homologous end joining (NHEJ) or more precise homology-directed repair (HDR). Because HDR is inefficient in non-dividing muscle cells, NHEJ-based strategies are more relevant for DMD applications. Several CRISPR approaches are being explored to restore the dystrophin reading frame, including exon deletion, splice-site modification, and frame-shifting [151]. However, effective delivery—primarily via AAV vectors—remains a major limitation, prompting exploration of non-viral methods such as lipid nanoparticles. Despite their potential, CRISPR-based therapies must overcome challenges related to off-target effects and immune responses but with the advantage of potentially requiring only a single treatment [152].

### 6.3. Storage Diseases

To date, the only approved treatment available for PD is enzyme replacement therapy (ERT), which consists of intravenous administration of recombinant human acid alpha-glucosidase [153]. The major therapeutic benefit of ERT is observed at the cardiac level, decreasing hypertrophy. Effects on growth delay have been documented too. The skeletal muscle response is comparatively modest, even at high doses [154]. Moreover, ERT does not cross the blood–brain barrier, so it is ineffective in treating neurological disorders. ERT has improved outcomes but has limitations, including immune reactions, incomplete tissue targeting, and the need for frequent lifelong infusions. These limitations have led to the exploration of new therapies independent or complementary to ERT including gene therapy, substrate reduction therapy (SRT) and pharmacological chaperone therapy (PCT) [155]. Gene therapy aims to provide a long-lasting correction by delivering a functional copy of the defective gene to the affected cells, restoring enzyme activity, and reducing glycogen accumulation [138]. Gene therapy with AAV vectors has been used in preclinical studies in PD, demonstrating promising results [156]. PCT is an alternative strategy that uses small molecules (chaperones) to stabilize the mutant enzyme and improve its folding and trafficking to lysosomes, thus enhancing residual enzymatic activity. Early studies showed that these molecules can modestly increase GAA activity in cells [157]. Some of these compounds also showed synergy when combined with ERT, improving the delivery and stability of the recombinant enzyme [158]. Another emerging approach is SRT, which aims to reduce glycogen production by inhibiting the muscle-specific enzyme that builds glycogen in muscle, called glycogen synthase 1. SRT can be used alone or in combination with ERT, resulting in lower glycogen accumulation, improved muscle pathology, and enhanced functional outcomes [159].

As regards MPS, two clinically available treatments are present: ERT (laronidase) and hematopoietic stem cell transplantation (HSCT). Both therapeutic approaches have been shown to improve quality of life and reduce disease-related morbidity, particularly when initiated early [160,161]. These approaches have also demonstrated beneficial outcomes in cardiac function and hypertrophy [97,162]. There are also side effects related to these therapies. ERT has limitations related to the blood–brain barrier, resulting in continued progression of neurological involvement despite improvements in systemic manifestations [163]. HSCT carries high morbidity and mortality, requires preparative chemotherapy, and carries a risk of graft failure [106]. Alternative therapeutic approaches have been explored to overcome these limitations. Preclinical gene therapy studies in MPS I have shown that restoring α-L-iduronidase activity can be effective. Significant challenges remain in maintaining long-term enzyme levels throughout the body and central nervous system, as well as in the use of certain vectors due to safety concern [161]. Gene therapy has the potential to enable low-level enzyme penetration across the blood–brain barrier [164]. Although it is still under investigation, if successful, this approach could provide a permanent, low-risk cure for MPS I. SRT is another approach under investigation for various MPS subtypes. This strategy aims to reduce the synthesis of GAGs, the natural substrates of the deficient enzyme, in order to restore balance between enzyme activity and GAG turnover [165]. Certain chemical inhibitors, such as Rhodamine B and Genistein, are able to cross the blood–brain and blood–cornea barriers, reaching tissues that are otherwise difficult to access. Research on this approach remains preclinical, and clinical trials are needed to evaluate potential side effects [166,167].

### 6.4. Mitochondrial Diseases

The management of MDs poses significant challenges due to the inability to conduct large-scale trials, which is a consequence of the rarity of these conditions. The delivery of therapy into the mitochondria introduces an additional layer of complexity. A group of MDs, predominantly those associated with cofactor metabolism disorders, demonstrated a positive response to etiologic treatments. Despite limited curative treatments, significant advancements have been made in the domains of metabolic supplementation, lifestyle interventions, and the development of targeted pharmacological, dietary, and genetic therapies [168]. Coenzyme Q10 (CoQ10) is a vital electron carrier and antioxidant that is frequently prescribed. The most substantial evidence of its efficacy is observed in cases of primary CoQ10 deficiency. Thiamine, biotin, and riboflavin have been shown to be highly effective in selected inborn errors of metabolism, particularly those pertaining to disorders of vitamin transport or cofactor synthesis. Notwithstanding the pervasive utilization of combined vitamin and antioxidant supplements, evidence substantiating their routine incorporation into the therapeutic regimens of MD populations remains scant [169,170]. The efficacy of gradual and individualized exercise therapy has been demonstrated in the improvement of quality of life, as well as in the promotion of mitochondrial biogenesis and oxidative phosphorylation [168]. The ketogenic diet has been demonstrated to be advantageous in cases of pyruvate dehydrogenase complex deficiency. Medium-chain triglycerides are subjects of investigation due to their potential to enhance mitochondrial metabolism [171]. Niacin and novel NAD+ precursors exhibited favorable safety profiles and outcomes. The endeavors undertaken to mitigate oxidative stress have yielded equivocal outcomes. Idebenone is the most well-established antioxidant therapy, particularly for cases of Leber hereditary optic neuropathy. In contrast, the efficacy of other redox-modulating agents has been variable or inconclusive [172]. Preserving mitochondrial membrane integrity and dynamics has emerged as another promising strategy. Agents targeting mitochondrial fusion, fission, and cristae structure—most notably elamipretide—have demonstrated encouraging preliminary results. The restoration of mtDNA synthesis and maintenance exemplifies a highly effective application of precision therapy in MD [173]. Deoxynucleoside supplementation and mitochondrial augmentation therapy are currently being explored, with good preliminary results. Genetic therapies represent the most innovative frontier in MD treatment. While gene replacement and RNA-based therapies for nuclear-encoded MD have shown success in preclinical models, translation to humans is constrained by tissue targeting and safety concerns. Conversely, allotopic expression and heteroplasmy-shifting approaches for mtDNA disorders have exhibited clinical efficacy [174,175]. Advancements in base editing, heteroplasmy modulation, and reproductive technologies, including mitochondrial replacement therapy, are poised to transform the therapeutic landscape for these disorders in the foreseeable future.

## 7. Conclusions

The concomitant occurrence of cardiomyopathies and growth delay has been documented in children affected by various inherited disorders. This phenomenon is attributable to shared genetic and molecular mechanisms. The variety of cardiac and growth impairment manifestations ranges from HCM and DCM to short stature. Recently, novel etiologic therapies have been approved, and others are currently under investigation. They have the potential to modify disease progression and represent the future direction for the treatment of these disorders. Preliminary genetic diagnosis and customized interventions are of paramount importance in maximizing outcomes and enhancing the quality of life for affected children.

## Figures and Tables

**Table 1:** Summary of the main genetic diseases associated with cardiomyopathy and growth impairment in children, highlighting the underlying mutations and altered biochemical pathways.

DISEASE	GENETIC MUTATIONS	BIOCHEMICAL PATHWAY	CARDIAC PHENOTYPE	GROWTH IMPAIRMENT
**RASopathies**	RAS/MAPK pathway genes (e.g., PTPN11, SOS1/2, KRAS, BRAF)	Hyperactivation of RAS/MAPK → dysregulated cell proliferation, differentiation, and survival [13]	- CHD and/or HCM (variable spectrum depending on genotype) [10] - Arrhythmias may occur	- Altered GH secretion and reduced GH peripheral response - Postnatal growth deceleration [9] - Most severe: Costello syndrome [29]
**Dystrophinopathies**	DMD gene (X-linked); frameshift → Duchenne in-frame → Becker	Truncated dystrophin → membrane fragility, altered calcium handling → ↑ apoptosis/necrosis [31]	- Progressive DCM in adolescence, modest LV dilation despite severe dysfunction, myocardial fibrosis - Arrhythmias common [39]	- Long-term glucocorticoid therapy - Mutations in the distal segment of the dystrophin gene (short stature even without steroids) [55]
**Pompe disease** **(GSD II)**	GAA gene (autosomal recessive)	Lysosomal acid α-glucosidase deficiency (<1% = IOPD; >1% = LOPD) → glycogen accumulation in lysosomes (cardiac and skeletal muscle dysfunction) [65]	CIOPD: severe HCM with rapid progression to DCM; conduction defects, arrhythmias, ischemia. NCIOPD: mild cardiac involvement (when present) [71]	IOPD: precocious puberty and feeding difficulties → accelerated bone age and reduced adult height LOPD: usually normal growth [82]
**Mucopolysaccharidoses**	Genes involved in degrading GAGs (mostly autosomal-recessive; MPS II is X-linked)	Enzyme deficiency → lysosomal GAG accumulation → inflammation, altered lipid signaling, apoptosis [85]	- Especially in MPS I, II, VI - Valvulopathy, HCM, arrhythmias, coronary artery disease [97]	- Progressive due to skeletal dysplasia (cartilage apoptosis, synovial hyperplasia) - Most severe: MPS IVA/VI - Mildest impact: MPS III [87]
**Mitochondrial diseases**	mtDNA or nuclear genes involved in mitochondrial function (e.g., NDUFS1/2, POLG, DNM1L)	Defective OXPHOS, mtDNA maintenance/expression, cofactor biosynthesis, lipid membranes, ATP production, ROS, calcium homeostasis, apoptosis [116]	- Concentric HCM - Possible primary DCM - LV noncompaction - Conduction defects (Kearns–Sayre syndrome) [128] - Arrhythmias (Barth, PPA2) [136] - Occasional WPW, valvulopathy [126]	- Poor nutrition, defective substrate use, energy deficiency, feeding difficulties - GH deficiency (mtDNA deletions, MELAS) - Short stature and low BMI [7]

BMI: Body Mass Index; CHD: congenital heart diseases; CIOPD: classical infantile-onset Pompe disease; DCM: dilated cardiomyopathy; GAGs: glycosaminoglycans; GH: growth hormone; GSD: glycogen storage diseases; HCM: hypertrophic cardiomyopathy; IOPD: infantile-onset Pompe disease; LOPD: late-onset Pompe disease; LV: left ventricle; mtDNA: mitochondrial DNA; MPS: mucopolysaccharidoses; NCIOPD: non-classical infantile-onset Pompe disease WPW: Wolff–Parkinson–White.

## Data Availability

No new data were created or analyzed in this study. Data sharing is not applicable to this article.

## Abbreviations

The following abbreviations are used in this manuscript:

CMPs	Cardiomyopathies
HF	Heart failure
DCM	Dilated cardiomyopathy
HCM	Hypertrophic cardiomyopathy
MAPK	Mitogen-activated protein kinase
NS	Noonan syndrome
CFCS	Cardio-facio-cutaneous syndrome
CHD	Congenital heart diseases
PVS	Pulmonary valve stenosis
NSML	Noonan syndrome with multiple lentigines
LVOTO	Left ventricular outflow tract obstruction
GH	Growth hormone
RhGH	Recombinant human growth hormone
DMD	Duchenne muscular dystrophy
BMD	Becker muscular dystrophy
Dp	Dystrophin
ECG	Electrocardiogram
LV	Left ventricle
CMR	Cardiac magnetic resonance
LGE	Late gadolinium enhancement
LVEF	Left ventricular ejection fraction
RV	Right ventricle
GSD	Glycogen storage diseases
PD	Pompe disease
GAA	Acid α-glucosidase
CIOPD	Classical infantile-onset Pompe disease
NCIOPD	Non-classical infantile-onset Pompe disease
LOPD	Late-onset Pompe disease
WPWs	Wolff–Parkinson–White syndrome
MPS	Mucopolysaccharidoses
GAGs	Glycosaminoglycans
MD	Mitochondrial diseases
MtDNA	Mitochondrial genome
MELAS	Mitochondrial Encephalomyopathy with Lactic Acidosis and ‘Stroke-like’ episodes
MERRF	Mitochondrial Encephalomyopathy with Ragged red Fibers
CPEO	Chronic Progressive External Ophthalmoplegia
KSS	Kearns–Sayre syndrome
BMI	Body Mass Index
AONs	Antisense oligonucleotides
AAV	Adeno-associated virus
NHEJ	Non-homologous end joining
HDR	Homology-directed repair
ERT	Enzyme replacement therapy
SRT	Substrate reduction therapy
PCT	Pharmacological chaperone therapy
HSCT	Hematopoietic stem cell transplantation
CoQ10	Coenzyme Q10
